# The Pattern of Use, Effectiveness, and Safety of Gadoteric Acid (Clariscan) in Patients Undergoing Contrast-Enhanced Magnetic Resonance Imaging: A Prospective, Multicenter, Observational Study

**DOI:** 10.1155/2021/4764348

**Published:** 2021-10-31

**Authors:** Won-Jin Moon, Young Ah Cho, Seok Hahn, Hye Min Son, Sung Koo Woo, Young Han Lee

**Affiliations:** ^1^Department of Radiology, Konkuk University Medical Center, Konkuk University School of Medicine, Seoul, Republic of Korea; ^2^Department of Radiology, Asan Medical Center, Seoul, Republic of Korea; ^3^Department of Radiology, Inje University Haeundae Paik Hospital, Busan, Republic of Korea; ^4^Department of Radiology, Yeungnam University Medical Center, Daegu, Republic of Korea; ^5^Department of Radiology, Dong-Eui Medical Center, Busan, Republic of Korea; ^6^Department of Radiology, Yonsei University College of Medicine, Seoul, Republic of Korea

## Abstract

**Objective:**

Contrast-enhanced MR (CE-MR) imaging is often required to improve lesion detection and characterization and to increase diagnostic confidence. This study aimed to evaluate the safety and effectiveness, as well as the use pattern, of the macrocyclic gadolinium-based contrast agent Clariscan in real-world clinical practice in Korea.

**Materials and Methods:**

This was a prospective, multicenter, observational study of patients undergoing CE-MR as part of routine clinical care at 6 university hospitals in Korea. Effectiveness was evaluated by determining diagnostic confidence and image quality; safety evaluation included the adverse event (AE) expression rate. Subgroup analyses were conducted by body regions of diagnosis (musculoskeletal, nervous system, others) and in pediatric patients (aged ≤7 years).

**Results:**

From October 2019 to September 2020, 1,376 subjects were included in the study. The mean volume of Clariscan used was 0.26 mL/kg (0.13 mmol/kg). In the overall study population and in each subgroup, diagnostic confidence increased after contrast enhancement with Clariscan. Overall, image quality was excellent in 72.5% of subjects and good-to-adequate in 27.2%. Clariscan was well tolerated (14 AEs occurred in 10 subjects); all AEs were of mild severity. Subgroup analyses showed that the mean dose of Clariscan used was ≥0.1 mmol/kg for nervous system-related diagnoses (e.g., brain) and ≤0.1 mmol/kg for musculoskeletal and pediatric-related diagnoses. All musculoskeletal and pediatric examinations were provided with a smaller package of 5 mL Clariscan. By body region of MR examination, the most common region was the nervous system in 69.0%, musculoskeletal system in 13.6%, and reproductive system in 4.9%.

**Conclusions:**

This study confirmed the use pattern of Clariscan and its excellent effectiveness and safety in the real-world clinical environment in Korea. The small-dose package indicated the possibility of increasing the convenience and efficiency of drug use.

## 1. Background

Contrast-enhanced magnetic resonance (CE-MR) imaging is often required to improve lesion detection and characterization and to increase confidence in diagnosis [1]. Gadoterate meglumine, a macrocyclic paramagnetic gadolinium-based contrast agent (GBCA) with high thermodynamic stability [2], has been available commercially in Europe since 1989. Since then, its use has been approved in more than 80 countries for numerous indications [3, 4]. It was approved by the US Food and Drug Administration (FDA) in March 2013 for use in the imaging of cerebral and spinal lesions and associated tissues with disrupted blood-brain barrier or abnormal vascularity in adults and children aged more than 2 years [5]. The macrocyclic structure of gadoterate meglumine provides greater chelate stability and a lower propensity to gadolinium release compared with other linear-structured GBCAs [6]. Since the first report of nephrogenic systemic fibrosis [7] and evidence of gadolinium deposition in the brain [8], there have been concerns regarding the appropriate and safe use of GBCA in patients [1]. Although macrocyclic GBCA is deemed as a safe choice, the selection and use of GBCA in the post-NSF era is expected to be more careful in terms of the type and dose of GBCA in clinical settings [9]. Recently, a new macrocyclic GBCA, Clariscan® (GE Healthcare), has become available in many countries. Clariscan has the same formulation of gadoteric acid as Dotarem® (Guerbet Laboratories). The safety and effectiveness of gadoterate meglumine have been evaluated in numerous previous studies [1, 10–12]. However, there is a lack of real-world clinical evidence regarding gadoterate meglumine and, in particular, Clariscan, in Korea. Therefore, the current study aimed to evaluate the effectiveness and safety, as well as the use pattern, of Clariscan in real-world clinical practice in Korea.

## 2. Materials and Methods

### 2.1. Study Design and Subjects

This prospective, multicenter, observational study was approved by the Institutional Review Boards of the participating centers. Written informed consent was obtained from patients.

The study included a total of 1,376 subjects who were administered Clariscan (0.5 mmol/mL, GE Healthcare) for MRI at 6 sites in Korea between Oct. 2019 and Sep. 2020.

Clariscan was administered in accordance with the guidelines and procedures of the individual participating institutions. Individuals of all ages were considered in this study. The exclusion of patients was based on the institution's criteria of contraindication of GBCA and the recommendation guidelines.

### 2.2. Assessment Variables

For demographic characteristics, gender, age, height, weight, body mass index (BMI), and type of referral were collected. For clinical characteristics, volume of contrast, use of automatic injector, field strength of MRI, previous imaging examinations, allergies, renal replacement, concomitant medications, and comorbidities were examined. Diagnosis of MR included provisional diagnosis and putative diagnosis. Body regions of MR examinations were classified by system organ class (SOC).

Effectiveness assessment was based on image quality and diagnostic confidence reported by local radiologists. Image quality for MRI and/or MRA images were assessed on a 4-point Likert scale: 1, poor, inadequate/blurring of the arterial segment; 2, fair, partial/inadequate arterial enhancement for confident diagnosis; 3, good, adequate arterial enhancement for confident diagnosis; and 4, excellent, excellent arterial enhancement for highly confident diagnosis. Diagnostic confidence was assessed using the most representative lesion for each patient. Before reviewing the CE-MR image, the radiologist was asked to document their level of confidence in making a diagnosis for the patient, with 5 categories each of 20% as a whole number between 0% and 100% based only on the nonenhanced image. After reading the CE-MR scan results, the radiologist was then asked to document their diagnostic confidence, using the same 5 categories each of 20% as a whole number between 0% and 100%. Results were used to calculate the change in diagnostic confidence by the local radiologist, before/after Clariscan use.

For safety assessment, the incidence of adverse events (AEs) occurring up to 7 days from Clariscan administration along with the number of subjects with AEs and number of events was presented. AEs were standardized by SOC and preferred term (PT) according to MedDRA. Status of radiological examinations including Clariscan at each site and satisfaction of the medical staff with Clariscan were collected as site information.

The primary study objective was to assess the pattern of use of Clariscan in MRI centers in Korea; the secondary objective was to evaluate the effectiveness and safety profile of Clariscan in real-life settings. To assess study objectives in specific populations, subgroup analyses were also conducted in patients who underwent MRI examination of the nervous system, musculoskeletal system, others, and pediatric patients (aged ≤7 years).

### 2.3. Statistical Analysis

Generally, summary statistics (mean, standard deviation, median, minimum, and maximum) were presented as continuous variables, and the number of subjects (*N*) and frequency (%) were presented as categorical variables. Demographic and clinical characteristics, body regions of MR examinations, quality of images, diagnostic confidence, and AEs were summarized by descriptive statistics. The incidence of AEs was presented with a 95% confidence interval. All analyses were performed using R version 3.5.1. software.

## 3. Results

### 3.1. Whole Population

**Figure 1:**
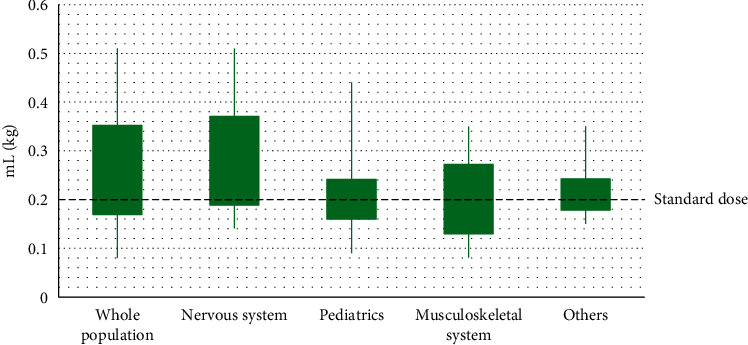
Volume use of Clariscan in the whole population and by subgroup.

Table 1 shows the demographic and clinical characteristics of the whole population (1376 subjects). Overall, 52.7% of patients were female, mean patient age was 52.6 years, and mean weight was 57.5 kg. The type of referral was routine in 1,323 subjects (96.2%) versus emergency in 53 subjects (3.9%). The mean volume of Clariscan used was 0.26 mL/kg (0.13 mmol/kg) (Table 1 and Figure 1). Automatic injectors were used for MR in 948 subjects (68.9%). MRI field strength was 3 T in 1,299 subjects (94.4%). In terms of previous imaging examinations, the most and least commonly used methods were MRI and single-photon emission computed tomography in 58.6% and 2.3% of subjects, respectively. The most common concomitant medications were angiotensin receptor antagonists (4.2%), and the most common comorbidity was hypertension (13.5%) followed by diabetes (8.7%).

**Figure 2:**
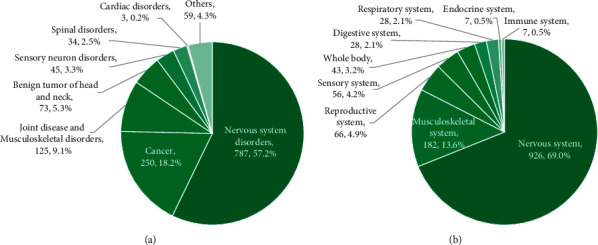
Distribution of (a) reasons for MR examination and (b) the body regions of MR examination in the whole study population (*N* = 1376).

Figure 2(a) shows the distribution of MR-related diagnoses. The most common reasons for MR examination were nervous system disorders (57.2%), cancer (18.2%), joint disorders (5.8%), and musculoskeletal disorders (3.3%).

Figure 2(b) shows the distribution of body regions of MR examinations by SOC. The most common body regions of MR examinations were the nervous system (69.0%), musculoskeletal system (13.6%), and reproductive system (4.9%).

**Figure 3:**
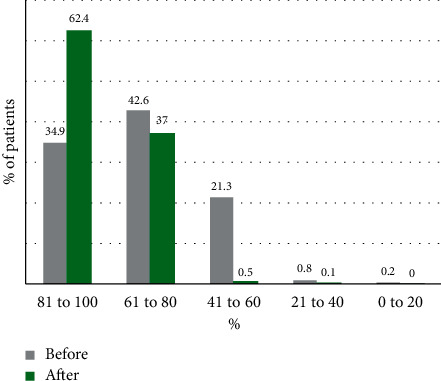
Diagnostic confidence before/after CE-MR in the whole study population (*N* = 1376).

**Figure 4:**
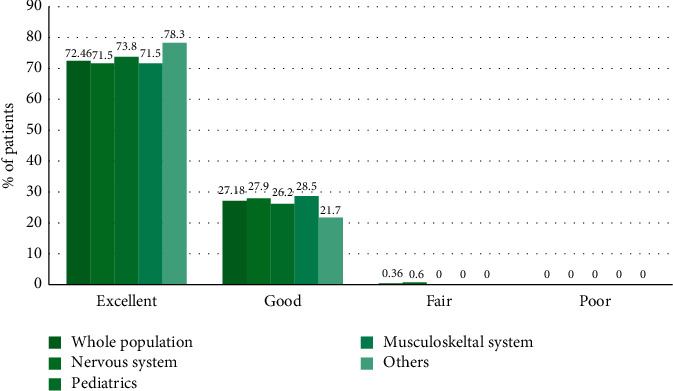
Image quality assessment after CE-MR in the overall population (*N* = 1376) and by subgroup.

For effectiveness evaluation, diagnostic confidence before and after Clariscan use and the quality of images were examined (Figure 3). Diagnostic confidence was “81 to 100%” in 454 subjects (32.99%) before use versus 859 subjects (62.43%) after use, indicating an increase in diagnostic confidence after contrast enhancement. Results from the assessment of image quality showed that the most frequent assessment was “excellent” in 72.5% of patients, followed by “good” (27.2%) and “fair” (0.4%); none of the images was assessed as “poor” (Figure 4).

The characteristics of AEs reported in the whole study population are shown in Table 2. By the time of onset, 9 events in 8 subjects (0.6%) were immediate AEs, and 5 events in 2 subjects (0.2%) were delayed AEs. All 14 AEs were of mild severity. A causal relationship to Clariscan was classed as certain in 1 subject (0.1%) and possible in 9 subjects (0.7%). By body region of MR examinations, the most common AEs were reported among individuals undergoing nervous system examination: 5 AEs in 4 subjects (0.3%). A total of 14 AEs were reported in 10 subjects. By SOC, the most common AEs were investigations and skin and subcutaneous tissue disorders, each in 4 subjects (0.3%), followed by general disorders and administration site conditions in 2 subjects (0.2%), and gastrointestinal disorders in 1 subject (0.1%) (Table 2). Specifically, by PT, these AEs were pruritus and oxygen saturation decreased, each in 4 subjects (0.3%); urticaria in 3 subjects (0.2%); and nausea, chills, and edema, each in 1 subject (0.1%) (Table 2).

### 3.2. Subgroups

Effectiveness, safety, and use patterns were also analyzed in the following subgroups: nervous system (*N* = 885), pediatric patients (aged ≤7 years; *N* = 200), musculoskeletal system (*N* = 148), and others (*N* = 143). Demographic and clinical characteristics of patients in the subgroups are shown in Supplementary Table 1.

The mean volume of Clariscan used was 0.28, 0.20, 0.18, and 0.21 mL/kg (0.14, 0.10, 0.10, and 0.11 mmol/kg) in the nervous system, pediatric, musculoskeletal system, and others subgroups, respectively (Figure 1 and Supplementary Table 1). By body region of MR examination, the most common body region was brain in 869 subjects (98.2%) in the nervous system group and 57 subjects (28.5%) in the pediatric group and spine in 41 subjects (27.7%) in the musculoskeletal system group (Supplementary Table 2). All examinations for the pediatric group and musculoskeletal group were provided with the smaller package of 5 mL Clariscan while the other examinations were with the standard package of 10 or 15 mL Clariscan.

The proportion of subjects for whom the radiologist had ≥60% diagnostic confidence before Clariscan use was 86.3% in the nervous system group and 83.1% in the musculoskeletal system group; the proportion of subjects with ≥60% diagnostic confidence after Clariscan use was 99.3% and 100.0%, respectively; in each group, the change in the number of subjects with ≥60% diagnostic confidence before and after Clariscan use was statistically significant (nervous system: *p* < 0.0001; musculoskeletal system: *p*=0.0026) (Supplementary Table 3 and Supplementary Figure 1). In the pediatric group, ≥60% diagnostic confidence was evident in 2.0% of patients before Clariscan use, and this increased significantly to 99.0% after Clariscan use (*p* < 0.0001) (Supplementary Table 3 and Supplementary Figure 1). While diagnostic confidence was increased after Clariscan use in all subgroups, a particularly sharp increase was observed in the pediatric group. Image quality assessment showed that almost all cases (≥99.4%) in all subgroups were rated as “Excellent” or “Good,” with none of the images in any subgroup being assessed as “Poor” (Figure 4).

By time of onset of all 14 AEs, the 9 immediate AEs were observed in the pediatric group (*n* = 5) and the nervous system group (*n* = 4), while the 5 delayed AEs were reported in the musculoskeletal system and connective tissue disorders group (*n* = 3) and others group (*n* = 2). By SOC and PT, skin and subcutaneous tissue disorders (urticaria, pruritus) were most commonly observed in each subgroup, with the exception of the pediatric group (no cases).

## 4. Discussion

The objectives of this study were to prospectively collect data on the use pattern, and to evaluate the effectiveness and safety, of Clariscan in real-world postmarketing clinical settings in Korea. The overall results showed that Clariscan was used at a mean volume of 0.26 mL/kg (0.13 mmol/kg) mainly for contrast enhancement in the examination of nervous system disorders and cancer. In the overall study population, an increase in diagnostic confidence was observed with Clariscan—the proportion of subjects with ≥60% diagnostic confidence was 73.3% before Clariscan use versus 99.4% after contrast enhancement with Clariscan. The quality of images was most commonly assessed by radiologists as “excellent contrast-enhancement” in 72.5% of subjects. Consistent with the results of the present study, a 2015 review of previous studies that evaluated the safety and efficacy of gadoteric acid also showed a high level of improvement in diagnosis upon the use of gadoteric acid, with good or excellent image quality reported in 95 to 100% of cases [13]. Similar findings were reported recently in a prospective, cross-sectional, multicenter, observational study, confirming that GBCAs are used appropriately in Europe for a wide range of indications [14]. The study demonstrated a significant increase in diagnostic confidence after GBCA use and confirmed the good safety profile of GBCAs, with comparable results for all agents used (Clariscan, Dotarem (gadoteric acid), Gadovist (gadobutrol), and ProHance (gadoteridol)) [14].

In a prospective, observational study of gadoterate meglumine in 35,499 patients (SECURE), the incidence of AEs was reported to be 0.1% [1]. An observational study in 1,631 children included in the SECURE study reported 1 AE (mild vomiting after an MRI) in a 2-year old child [15]. In a German postmarketing surveillance (PMS) study on 84,621 patients who were treated with gadoteric acid (Dotarem), the incidence of AEs was 0.4% [3]. A Japanese PMS study in 3,444 patients treated with gadoterate meglumine (Magnescope® in Japan, Dotarem® in other countries) reported an AE incidence of 0.9% [12]. The incidences of AEs were 0.8%, 0.3%, and 0.3% in PMS studies conducted in 3 European countries (France, Belgium, and Switzerland), France, and Germany, respectively [10]. In the present study, 14 AEs occurred in 10 subjects, resulting in an AE incidence of 0.7%, which is similar to, or lower than, the incidences reported in the previous studies. All of the AEs were mild in severity, and a causal relationship to Clariscan was classified as “certain” in 1 subject (0.1%) and “possible” in 9 subjects (0.7%). In addition, the majority of AEs occurred within 24 hours, that is, immediately after Clariscan injections. As with the previous studies, no severe AEs were reported [16, 17]. In the current study, 2.5% of children experienced AEs of mild severity, which was also comparable to the previous studies in terms of reaction, severity, and outcome [13, 18, 19]. Of the AEs reported in children in the present study, “oxygen saturation decreased” was considered by the investigators to be attributable to sedation as all of the children underwent MRI examinations under sedation.

Based on these real-world results, Clariscan is considered to be effective and safe for use as an MRI contrast. Unlike conventional agents that are supplied in mid to large volumes of ≥10 mL, Clariscan is supplied in a small 5 mL package. Hence, it is notable that, in the subgroup analyses, a smaller volume of Clariscan was used in pediatric patients and those with musculoskeletal and connective tissue disorders in routine clinical practice compared with doses normally used for other diseases in adults. Higher mean doses of Clariscan used in the subgroup of patients with nervous system disorders reflect a large number of brain CE-MR images. In pediatric patients, the lower mean volume of Clariscan was associated with a statistically significant increase in diagnostic confidence: ≥60% diagnostic confidence was evident in 2.0% of patients before Clariscan use, increasing to 99.0% after Clariscan use (*p* < 0.0001).

The study is limited in that it employed descriptive statistics only. However, the study is considered meaningful because it is the first prospective study conducted in Korea on the use patterns, effectiveness, and safety of Clariscan in routine clinical settings. An additional strength of this study is the classification and evaluation of Clariscan use in children, which highlights the need to further study the use of GBCAs in special populations such as infants and children.

## 5. Conclusion

This study identified use patterns of Clariscan and confirmed the favorable effectiveness and safety profiles of Clariscan in routine clinical settings in Korea. In a noncomparative comparison with previous studies, no notable differences were observed between Clariscan and conventional agents in terms of effectiveness and safety. Furthermore, the study indicated the potential for the small-volume package of Clariscan to contribute to increased convenience and effectiveness in drug use.

## Figures and Tables

**Table 1:** Demographics and clinical characteristics of patients in the whole study population.

Category	*N* = 1376
	*N* (%)
Gender	
Male	651 (47.3)
Female	725 (52.7)
Age (years), mean ± SD	52.6 ± 25.2
Height (cm), mean ± SD	153.5 ± 27.0
Weight (kg), mean ± SD	57.5 ± 21.0
BMI (kg/m^2^), mean ± SD	22.9 ± 4.0

Referral	
Routine	1323 (96.2)
Emergency	53 (3.9)
Follow-up	0 (0.0)
MR examination	*N* (%)
Volume use (mL/kg), mean ± SD (median, min, max)	0.26 ± 0.09 (0.21, 0.08, 0.51)

Automatic injector used	
Yes	948 (68.9)
No	428 (31.1)

Field strength of MRI	
1.5 T	77 (5.6)
3 T	1299 (94.4)
Other	0 (0.0)

Previous imaging examinations^∗^	
MRI	806 (58.6)
CT	445 (32.3)
Ultrasound	273 (19.8)
PET	94 (6.8)
SPECT	31 (2.3)
None	288 (20.9)
Medical history	*N* (%)

Allergies	
Yes	40 (2.9)
No	1336 (97.1)

On renal replacement	
Yes	4 (0.3)
No	1372 (99.7)

Concomitant medications^∗^	
Beta-blocker	41 (3.0)
Vasoactive substances	13 (0.9)
ACE inhibitors	6 (0.4)
Angiotensin receptor antagonist	58 (4.2)
None	1282 (93.2)
Comorbidities	*N* (%)
No	996 (72.4)
Yes	380 (27.6)

Types of comorbidities^*∗*^	
Hypertension	186 (13.5)
DM	119 (8.7)
CNS disorder (seizures/epileptics/convulsion)	40 (2.9)
Heart failure	22 (1.6)
History of kidney disease	15 (1.1)
History of CM ADR	13 (0.9)
Liver disease/liver transplantation/liver surgery	13 (0.9)
Allergic disorder	4 (0.3)
Asthma	3 (0.2)
History of kidney surgery	2 (0.2)
Others	134 (9.7)

^*∗*^Multiple responses.

**Table 2:** Characteristics and symptoms (by SOC and PT) of adverse reactions in the whole study population.

Adverse reactions	*N* = 1376			
	No. of subjects	No. of events	Incidence rate (%)	95% CI (lower, upper)
Type of adverse reaction				
Immediate	8	9	0.58	(0.002, 0.010)
Delayed	2	5	0.15	(−0.001, 0.004)

Severity				
Mild	10	14	0.73	(0.003, 0.012)
Moderate	0	0	0	—
Severe	0	0	0	—

Causality				
Certain	1	2	0.07	(−0.001, 0.002)
Likely	0	0	0	—
Possible	9	12	0.65	(0.002, 0.011)
Unlikely	0	0	0	—
Unassessable	0	0	0	—

Body region of MR exam				
Immune system	1	1	0.07	(−0.001, 0.002)
Musculoskeletal system	1	3	0.07	(−0.001, 0.002)
Nervous system	4	5	0.29	(0.000, 0.006)
Sensory system	1	2	0.07	(−0.001, 0.002)
Vascular system	1	1	0.07	(−0.001, 0.002)
Whole body	2	2	0.15	(−0.001, 0.004)
Total	**10**	**14**	**0.73**	**(0.003**, **0.012)**

Adverse reactions by SOC/PT^*∗*^	No. of subjects	No. of events	Incidence rate (%)	95% CI (lower, upper)

Gastrointestinal disorders	1	1	0.07	(−0.001, 0.002)
Nausea	1	1	0.07	(−0.001, 0.002)
General disorders and administration site conditions	2	2	0.15	(−0.001, 0.004)

Chills	1	1	0.07	(−0.001, 0.002)
Oedema	1	1	0.07	(−0.001, 0.002)
Investigations	4	4	0.29	(0.000, 0.006)
Oxygen saturation decreased^∗∗^	4	4	0.29	(0.000, 0.006)
Skin and subcutaneous	4	7	0.29	(0.000, 0.006)

Tissue disorders				
Pruritus	4	4	0.29	(0.000, 0.006)
Urticaria	3	3	0.22	(−0.0003, 0.005)
Total	**10**	**14**	**0.73**	**(0.003**, **0.012)**

^*∗*^PT: preferred term; SOC: system organ class, MedDRA (Ver. 20.1). ^∗∗^All cases registered in MR examinations under sedation.

## Data Availability

The datasets used and/or analyzed during the current study are available from the authors on reasonable request.

## Supplementary Materials

Supplementary Materials Supplementary Materials. Supplementary Table 1. demographics and clinical characteristics of patients (*N* = 1376) by subgroup. Supplementary Table 2. main body regions of MR examination by subgroup. Supplementary Table 3. diagnosis confidence before/after CE-MR by subgroup. Supplementary Figure 1. diagnostic confidence before/after CE-MR by subgroup: (a) nervous system, (b) pediatrics, (c) musculoskeletal system, and (d) others ().
