# Supplementary material for: The Pattern of Use, Effectiveness, and Safety of Gadoteric Acid (Clariscan) in Patients Undergoing Contrast-Enhanced Magnetic Resonance Imaging: A Prospective, Multicenter, Observational Study
Source: Contrast Media Mol Imaging. 2021 Oct 31;2021:4764348. doi: 10.1155/2021/4764348 (PMC8572637; doi:10.1155/2021/4764348)
Supplement: Supplementary Materials — Supplementary Table 1. demographics and clinical characteristics of patients (N = 1376) by subgroup. Supplementary Table 2. main body regions of MR examination by subgroup. Supplementary Table 3. diagnosis confidence before/after CE-MR by subgroup. Supplementary Figure 1. diagnostic confidence before/after CE-MR by subgroup: (a) nervous system, (b) pediatrics, (c) musculoskeletal system, and (d) others (). [file 4764348.f1.docx]

**The pattern of use, effectiveness, and safety of gadoteric acid (Clariscan) in patients undergoing contrast-enhanced magnetic resonance imaging: a prospective, multicenter, observational study**

**Supplementary Table 1**. Demographics and clinical characteristics of patients (N=1376) by subgroup

| **Category** | **Nervous system** | **Pediatrics** ^a^ | **Musculoskeletal system** | **Others** |
| --- | --- | --- | --- | --- |
| No. of patients | N=885 | N=200 | N=148 | N=143 |
| Gender, n (%) |  | | | |
| Male | 422 (47.7) | 99 (49.5) | 75 (50.7) | 55 (38.5) |
| Female | 463 (52.3) | 101 (50.5) | 73 (49.3) | 88 (61.5) |
| Age (years) | 64.1±14.1 | 2.8±2.0 | 50.2±18.6 | 53.4±14.8 |
| Height (cm) | 163.7±9.3 | 93.4±16.7 | 165.3±8.4 | 162.6±9.0 |
| Weight (kg) | 64.9±11.7 | 14.6±4.8 | 67.2±12.5 | 62.5±12.6 |
| Body mass index (kg/m^2^) | 24.1±2.9 | 16.4±1.9 | 24.5±3.6 | 23.5±3.4 |
| Volume use (mL/kg) | 0.28±0.09 | 0.20±0.04 | 0.18±0.09 | 0.21±0.03 |

Data are shown as mean±SD unless stated otherwise.

^a^ Patients aged ≤7 years.

**Supplementary Table 2**. Main body regions of MR examination by subgroup

| **Body Regions** | **N (%)** |
| --- | --- |
| **Nervous system (N=885)** | |
| Brain | 869 (98.2) |
| Internal auditory canal | 10 (1.1) |
| Brachial plexus | 2 (0.2) |
| Spine | 2 (0.2) |
| Foot | 1 (0.1) |
| Head and neck | 1 (0.1) |
| **Pediatrics (N=200)** | |
| Brain | 57 (28.5) |
| Whole body | 43 (21.5) |
| Eye | 27 (13.5) |
| Spine | 13 (6.5) |
| Digestive system | 12 (6.0) |
| Knee | 7 (3.5) |
| Lymphatic vessels | 7 (3.5) |
| Others | 34 (17.0) |
| **Musculoskeletal system (N=148)** | |
| Spine | 41 (27.7) |
| Shoulder | 34 (23.0) |
| Ankle | 19 (12.8) |
| Pelvis | 12 (8.1) |
| Hip joint | 8 (5.4) |
| Foot | 6 (4.1) |
| Others | 28 (18.9) |
| **Others (N=143)** | |
| Breast | 45 (31.5) |
| Sensory system | 28 (19.6) |
| Respiratory system | 25 (17.5) |
| Digestive system | 16 (11.2) |
| Endocrine system | 6 (4.2) |
| Others | 23 (16.1) |

**Supplementary Table 3**. Diagnosis confidence before/after CE-MR by subgroup


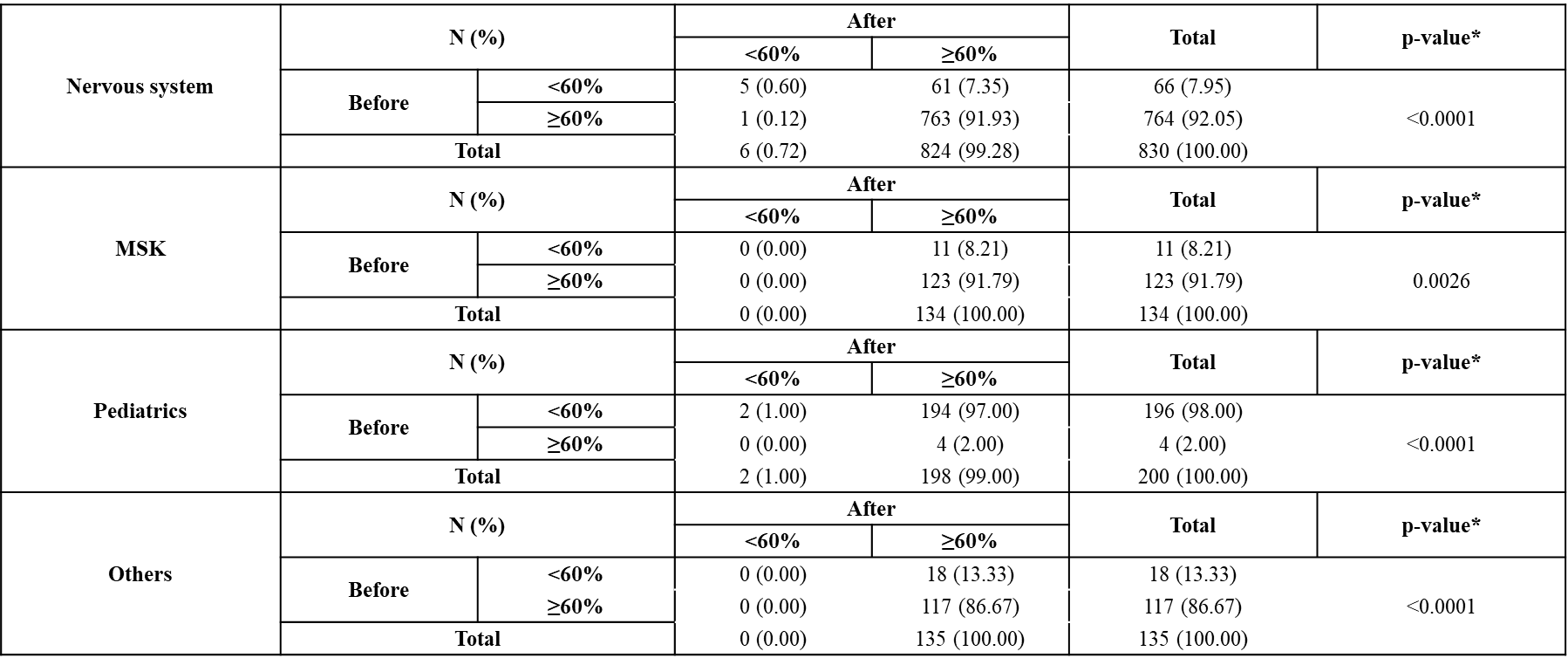


*McNemar’s test

**Supplementary Figure 1**. Diagnostic confidence before/after CE-MR by subgroup: (a) nervous system, (b) pediatrics, (c) musculoskeletal system, (d) others

(a)

(b)

(c)

(d)
